# STUDYING BIOLOGICAL AGING AND GEROSCIENCE IN HUMANS

**DOI:** 10.1093/geroni/igac059.1346

**Published:** 2022-12-20

**Authors:** Anne Newman

**Affiliations:** University of Pittsburgh, Pittsburgh, Pennsylvania, United States

## Abstract

For geroscience to impact human health, its scientific underpinnings observed in animal models must also be relevant to humans, and therapies grounded in geroscience principles must be translatable to humans. Given the heterogeneity of human aging, chronological age alone is not a sufficient indicator of susceptibility to specific diseases, disabilities, or death, nor does it provide insights into the likelihood of benefiting from specific geroscience-inspired approaches. Bringing these metrics to large epidemiological studies and clinical trials has provided a number of important insights. For example, indices developed from aggregates of biomarker, physiological and clinical data have been shown to perform well in terms of predicting healthy aging, specific aging-related traits and mortality. Functional or composite outcomes have been favored for capturing health span in translational trials.

